# Are Toxic Butterflies More Easily Detected by Human ‘Predators’?

**DOI:** 10.1002/ece3.73357

**Published:** 2026-04-06

**Authors:** Marilia Fernandes Erickson, Donald James McLean, Marie E. Herberstein

**Affiliations:** ^1^ School of Natural Sciences, Faculty of Science and Engineering Macquarie University Sydney New South Wales Australia; ^2^ Centre for Taxonomy and Morphology Leibniz Institute for the Analysis of Biodiversity Change Hamburg Germany; ^3^ Department of Biology University of Hamburg Hamburg Germany

**Keywords:** antipredator defence, aposematism, chemical defence, defensive colour, detection time, signalling

## Abstract

Butterfly colours often signal unprofitability due to toxicity to predators, with more conspicuous colour signals increasing predator avoidance learning. These signals can be on the dorsal and/or ventral wing surfaces, which are more visible during flight or at rest respectively. Here we are interested in the relationship between toxicity and conspicuousness in Australian butterflies. We use detectability as a proxy for conspicuousness and test whether toxic butterflies are more detectable on both the dorsal and ventral wing sides using a computer game where humans hunt butterflies against multiple backgrounds. The game was played by over 1700 players who collectively reacted to over 67,000 images of butterflies. Toxicity was assessed using *Daphnia* mortality assays on over 420 individuals from 61 species. Specifically, we tested (1) if the detectability of dorsal or ventral butterfly wings was related to toxicity, and (2) if butterflies that were more toxic had similar dorsal and ventral detectability. For dorsal surfaces, we found that the more toxic butterflies were detected faster than less toxic butterflies, whereas there was no relationship between toxicity and detection time for ventral surfaces. These findings are consistent with previous work that suggests aposematic signals are particularly relevant when animals are in motion, since the dorsal wing surface of butterflies—the more detectable side—is usually visible during flight rather than at rest. We also found that more toxic butterflies were more similar between dorsal and ventral detection, suggesting that greater levels of toxicity are communicated both ventrally and dorsally.

## Graphical abstract

Aposematic signals are often thought to be conspicuous and stable across environments. Yet, butterflies can have distinct colours on their dorsal and ventral sides which contradict this prediction. We found that despite toxic butterflies having similar detectability on both sides, only dorsal detectability positively correlated with toxicity.

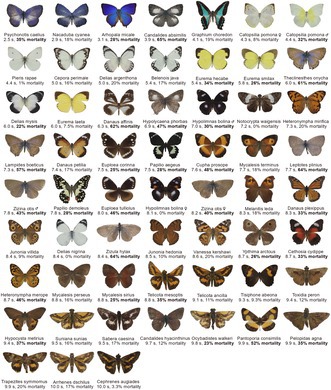

## 1 Introduction

Colourful animals are often accompanied by secondary defences such as toxins, a strategy known as ‘aposematism’ (Kikuchi et al. 2021). Defensive toxins render animals unpalatable to—or impose a fitness cost on—predators (Marples et al. 2018). If this unpleasant stimulus is coupled with distinctive colouration, aversive learning in predators can be elicited (Roper and Wistow 1986). After the initial learning process, predators avoid toxic and conspicuous morphs, resulting in a fitness benefit for the aposematic prey. Unlike crypsis, which is most effective when an animal is not moving (Ioannou and Krause 2009), aposematism can provide protection during movement and hence supports the exploitation of more resources (Speed et al. 2010). Aposematic animals benefit from promptly identifiable conspicuous patterns (Stevens and Ruxton 2012), in that conspicuousness reduces the time required to identify aposematic prey (Gittleman and Harvey 1980) and increases the retention time of aversive memories (Roper and Redston 1987). Conspicuousness, however, is highly dependent on the background environment—for example, a brown rabbit is cryptic against a brown background but conspicuous against snow (Endler 1978). Conversely, aposematic animals would gain maximum benefit from being conspicuous across multiple visual backgrounds (Arenas et al. 2014).

Although conspicuousness is used to describe aposematic signals, some nondefended or less defended animals have evolved similar colouration and patterns as defended models, a phenomenon called ‘mimicry’, which complicates the categorisation of aposematic animals. Additionally, conspicuous colours can also occur in other contexts, such as other defensive strategies, including disruptive coloration, deimatic displays (Troscianko et al. 2017; Umbers et al. 2015), and, in a sexual context, to attract mates. These conspicuous signals may be constantly advertised, or hidden and revealed, during specific behavioural displays—such as in the dewlap of lizards (Font and Rome 1990).

Butterflies are a well‐researched group in terms of aposematism as many species are conspicuous and chemically defended, making them a particularly interesting group to understand the evolution of toxicity relative to conspicuousness. Additionally, the two different wing surfaces (i.e., dorsal and ventral), may evolve distinct colour patterns with different functions for either surface (Oliver et al. 2009; Wee and Monteiro 2017). Depending on butterfly posture, ventral and dorsal colour wing surfaces are more visible during rest or flight respectively. Usually, butterflies rest with wings closed, exposing their ventral surface, although, while basking, butterflies can adjust wing angles to regulate heat absorption (Barton et al. 2014) and consequently may exhibit dorsal colours even when still. The dorsal side is exposed during flight due to wing flapping, and can communicate sexual signals, only visible at certain angles during courtship flight (e.g., White et al. 2015). This form of signal partitioning is not, however, universal in butterflies and some well‐known aposematic species (e.g., *Danaus, Heliconius*) have similar wing patterns on both sides.

Here we are interested in testing whether toxic butterflies are more conspicuous than nontoxic butterflies and whether the detectability of dorsal and ventral surfaces differs. We used detection time by human ‘predators’ as a proxy for conspicuousness: butterflies that are located more quickly against a background are considered more conspicuous, while more cryptic butterflies take longer to detect. We predict that toxic butterflies will be more conspicuous on their ventral side, so they are protected during rest and flight, and that toxic butterflies should have similar detection times on the dorsal and ventral surfaces to display a consistent signal while in motion. We address these questions by combining a comprehensive assessment of butterfly toxicity in 61 Australian butterfly species and butterfly images from a high‐quality database (Erickson 2025; Erickson et al. 2025), with measures of butterfly detection time derived from human responses to butterflies against different backgrounds in an online game.

## 2 Methods

### 2.1 Butterfly Collection

The toxicity data in this paper are shared with Erickson et al. (in prep) (59 species) and (Erickson 2025) (*Zizina otis* data; randomly selecting 12 assays out of the data set). Additional toxicity assays were conducted for *Zizula hylax* in the present paper. In total, we used 61 butterfly species that were collected from Cairns, Brisbane and Sydney between August 2022 and November 2023 and were represented by at least three individuals. From these, we produced an average toxicity score based on *Daphnia* assays (Table S1). Other than the minimum of three individuals, no other criteria were used to select these species, which represented a range of cryptic looking and aposematic looking species. In the field, we captured the animals using an entomological net and immediately placed them in a cooler bag to anaesthetise them and prevent dehydration. They were later euthanised by freezing and they were kept frozen until chemical extractions were conducted, although they were temporarily removed from the freezer for labelling, transport and photography (always in cooler bags with ice).

### 2.2 Toxicity Assays

To estimate butterfly toxicity, we used *Daphnia* mortality assays based on several previous studies (Arenas et al. 2015; Binns et al. 2025; Medina et al. 2020). Despite *Daphnia* not being predators, they are widely used as a proxy for toxicity in multiple taxa (Arenas et al. 2015; Aslam and Nedvěd 2024; Binns et al. 2025; Erickson 2025; Guilhermino et al. 2000; Harmon and Mousseau 2009; Medina et al. 2020; Mora‐Castro et al. 2021). Experimental comparisons across vertebrate and invertebrate laboratory models of toxicity further show that *Daphnia* exhibit particularly high sensitivity to many toxins relative to other test organisms and that physiological responses to toxins may be conserved across evolutionary lineages (Hayot et al. 2025). In addition, by choosing a toxicity model that has not evolved in contact with butterfly toxins, we are confident that *Daphnia* have not evolved resistance to the chemicals extracted. If we were to use predators that are in constant contact with some of the study species, they may have developed resistance to some chemicals but not others, biasing our results. This is of particular importance since we are working with a wide variety of species (~60).

In brief, butterfly bodies were homogenised with methanol, then the supernatant was removed, evaporated in a vacuum concentrator and rehydrated to the concentration of 0.008 g/mL. We conducted from three to 13 assays (depending on the availability of butterflies) for 61 species, totalling 423 assays. We combined 0.5 mL of butterfly stock with 10 *Daphnia magna* for each assay and counted the number of dead *Daphnia* after 4 h. This number of *Daphnia* was chosen so as not to crowd the vial. To control for potential effects of the extraction protocol, we included two solvent controls: a water control and a methanol control. For these controls, vials containing either Milli‐Q water or 99% methanol were subjected to the same vacuum concentration process and subsequently rehydrated with Milli‐Q water (*n* = 18 assays per control). The methanol control was included to verify that residual methanol did not contribute to *Daphnia* mortality if the solvent was not fully evaporated during vacuum concentration. In addition to the solvent controls, we prepared extracts from cricket ( *Acheta domesticus* ) and cockroach ( *Nauphoeta cinerea* ) following the same extraction protocol used for butterflies, to estimate baseline mortality expected from nontoxic solutions (*n* = 10 assays each).

### 2.3 Butterfly and Background Images

**FIGURE 1:**
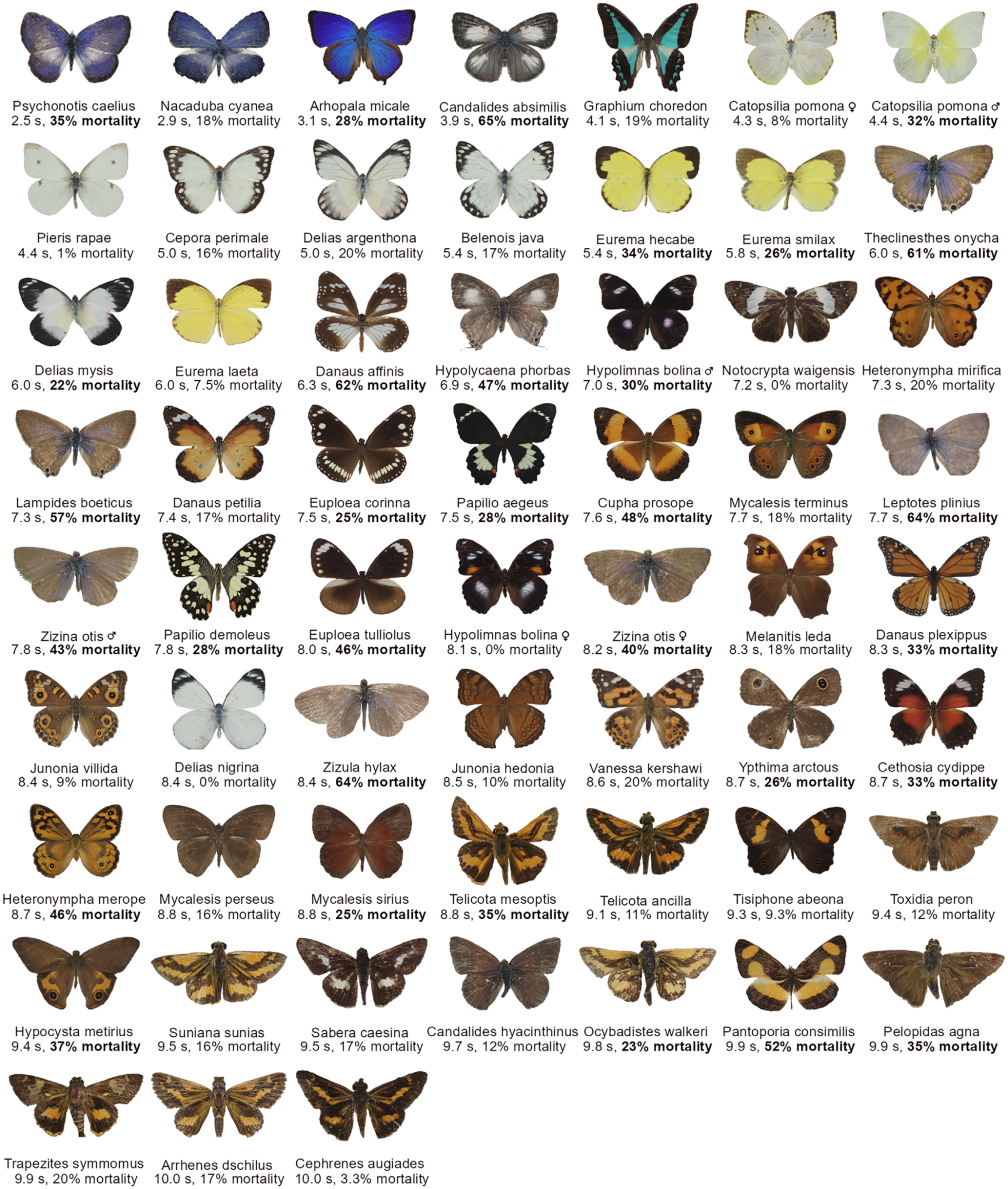
Dorsal images ordered by increasing mean detection time on most cryptic background; ordered from left to right and top to bottom. Individual captions show species name, sex for sexually dimorphic forms, detection time and mean *Daphnia* mortality for each morphospecies. Bold ‘mortality’ scores indicate the species mean mortality (toxicity) was higher than single assay of the controls (cricket, cockroach, milliq water and methanol) (> 20%).

**FIGURE 2:**
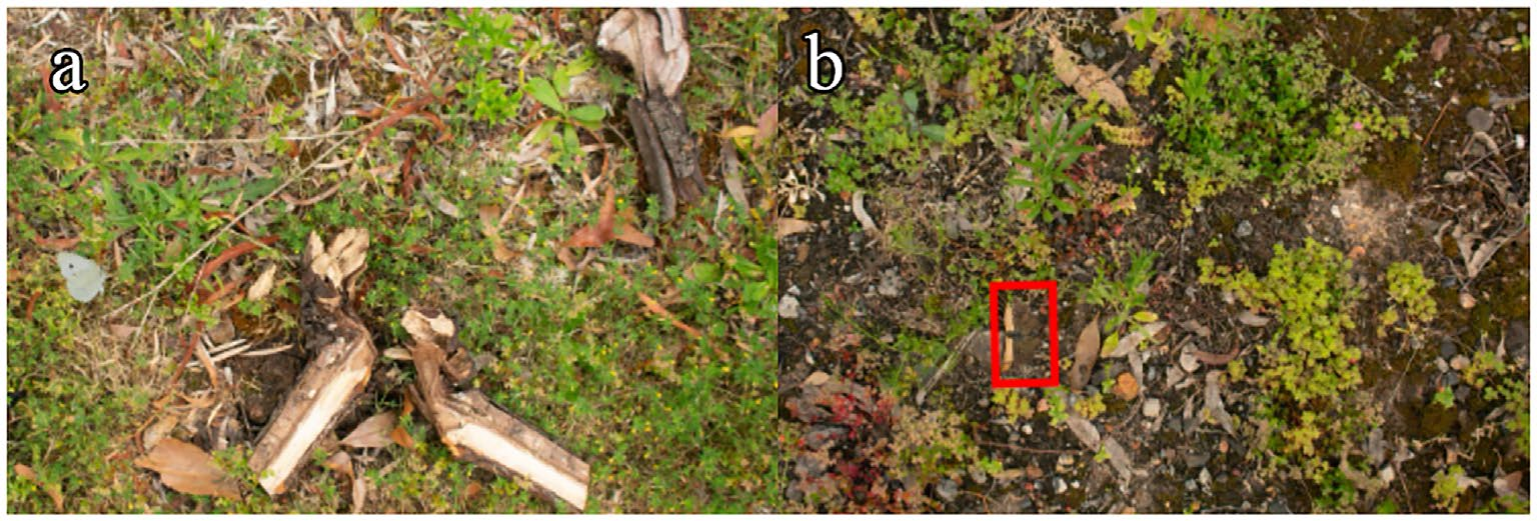
Example images of the game. (a) Image of a ventral pattern of a cabbage white butterfly on grass background (left side of the image). (b) Image of a dark branded swift on a moss background highlighted by a red square after the 10 s have passed and butterfly was not identified.

The images of the butterflies used to create this game were obtained from the *OZButterflies* database, and reflectance for wing measurements is available at the same source (Erickson et al. 2025). We used one dorsal and one ventral photograph for each species. The dorsal side was presented with wings open; (Figure 1) the ventral side with wings closed (Figure 2). The photos were calibrated for white balance using the white standard of the ColorChecker (X‐rite) passport and saved in TIFF format with Imaging Edge (Sony). We used the band aid tool in Adobe Photoshop to correct wing imperfections (e.g., tears and missing scales), then erased the background. If butterfly wings on one side (left or right) were damaged beyond repair, we copied and pasted and inverted the wing from the opposite side to generate an image of a complete butterfly. As most wings in the database were photographed separated from the butterfly bodies, we edited the images to re‐assemble the components into natural positions. Five of our species were missing dorsal images in the database. Images were saved in PNG format to allow for transparent backgrounds. To reduce the effect of image size on conspicuousness, images were scaled so that the area of the butterfly, excluding the background, was as close to 30,000 pixels as possible. In this way, we standardise the detection time of butterfly colour pattern independently of size, though we could not control for screen size of the users to take butterfly size into account statistically. For butterfly species that were obviously sexually dimorphic on the dorsal side (e.g., *Hypolimnas bolina*), we included separate dorsal images of males and females (*n* = 3 species). In total we had 61 species (plus three male/female morphotypes, totalling 64 morphotypes), from which we were able to use 59 dorsal and 61 ventral phenotypes totalling 120 possible butterfly images. For simplicity, in the remainder of the paper, we consider male–female morphotypes separately.

Thirty different backgrounds (background images available in the GitHub repository) were photographed using a Nikon D5200. The number of backgrounds represents the number of rounds in a game, which was designed to last a maximum of 5 min (10 s per image); this way each game showed all backgrounds once, but the butterfly images would vary for each game (randomly selecting from the 120 possible images). The only modification to background images was an adjustment of the white balance (using an X‐Rite ColorChecker) and exposure to match the brightness of butterfly photographs manually. We deliberately photographed backgrounds outside the collection sites of butterflies to standardise background images to general natural backgrounds. Despite not being the backgrounds of the actual collection sites, natural backgrounds such as leaves, tree bark and stones are not known to show major reflectance differences (Gutiérrez et al. 2025; Morimoto et al. 2022). Therefore, we expect that the nontoxic butterflies used in the experiment would be reasonably well suited to camouflage on at least one of the 30 background images provided, whereas aposematic species are considered to be conspicuous on multiple backgrounds (Spaniol et al. 2020) and should stand out in several of them. In our main analyses, we used only the detection time of the most cryptic background, assuming that nontoxic butterflies would evolve some kind of camouflage (see below).

### 2.4 Detection Time

We estimated butterfly conspicuousness, by creating a computer game to measure the time human participants required to detect a butterfly image against the image of a natural background. This method was inspired by similar games by Troscianko et al. (Troscianko et al. 2013, 2017, 2021). Humans are typically trichromats and have different visual systems to major butterfly predators (birds, lizards, spiders, etc.). One of the major differences is the lack of UV receptor. Indeed, several of the butterflies used in our study reflected UV light, but since UV scatters easily at a distance we believe humans are still good proxy‐predators for ‘hunting’ butterflies at a distance. The project did not require ethics approval as it was assessed as ‘low risk’ by the Macquarie University Human Ethics Committee, and no personal data were collected from participants.

**FIGURE 3:**
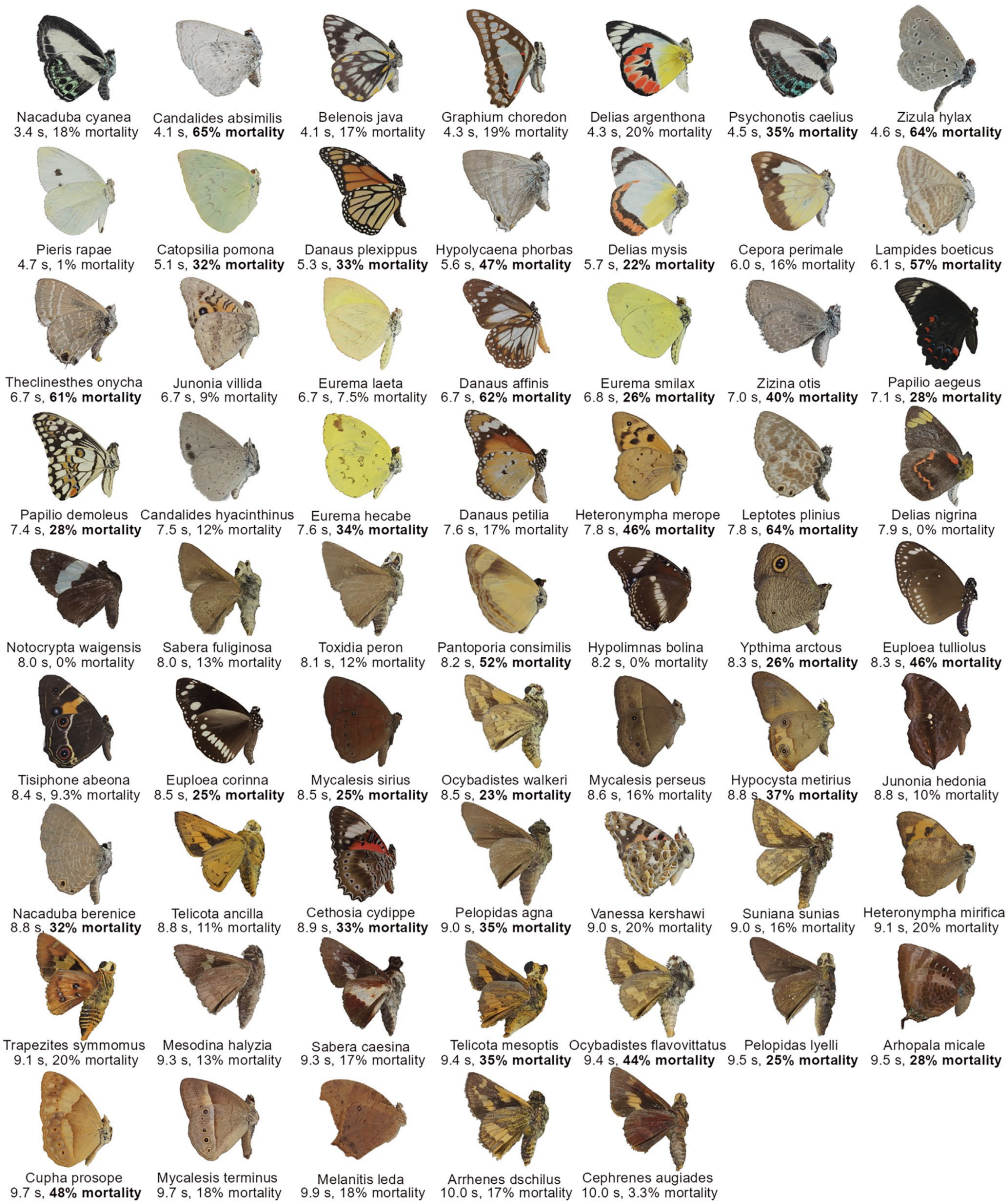
Ventral images ordered by increasing mean detection time on the most cryptic background, ordered from left to right and from top to bottom. Individual captions show species name, detection time and mean *Daphnia* mortality for each morphospecies. Bold ‘mortality’ scores indicate the species mean mortality (toxicity) was higher than single assay of the controls (cricket, cockroach, milliq water and methanol) (> 20%).

The game consisted of 30 rounds. In each round, a randomly selected butterfly image (out of the possible 120) was placed at a random location and orientation against a randomly selected background (out of the possible 30 backgrounds, though all backgrounds were used during a single game, albeit in a random order). The participants were instructed to click on the butterfly as quickly as possible. If they correctly clicked on the butterfly, the game proceeded to the next round. If the click was in an incorrect location, participants could try again until 10 s had passed. Upon failure to locate the butterfly within 10 s, the location of the butterfly was revealed, and the next round started (Figure 3). Participants could play the entire game (30 rounds) multiple times, with different butterflies and the backgrounds presented in a different order each time. To see if player replays influenced the detection time, we fitted a linear model to detection time and number of games played, with user ID as a random effect, for players that played at least twice. Players improved by only 0.006 s per game, with a marginal R^2^ of 0.001, meaning that learning due to replays only explained about 0.1% of the variation in the data set, so we considered it negligible.

For each round we recorded the butterfly species, the type of background image, the location and orientation of the butterfly image, the location and time of user clicks, whether the clicks were hits or misses, and if a timeout occurred. The game was implemented as a web application and was freely available to play (https://mferickson.github.io/findbutterfly/). We advertised the game on social media to recruit users. No personal information was collected, but we recorded anonymised user IDs so we were able to determine the number of distinct users. We excluded responders who found fewer than three butterflies as we assumed they did not understand the instructions properly. The game data were downloaded at 11 pm on the 26th of February 2025 (GMT +11). At this time the game had been played 2751 times comprising 67,062 rounds by 1716 users. Users were not required to complete games, in which case we used all completed butterfly scores. On average, players scored 24.4 butterflies per game.

We estimated detectability for each butterfly image by calculating the average detection time against each background (detection failures were included as 10 s). For each butterfly, we calculated mean detection time against every background, to identify the background against which it was mostly difficult to spot; mean detection time against that background was then used as the measure of detectability for that butterfly. The reason this metric was chosen is because we assumed cryptic species would have evolved for maximum crypsis on specific backgrounds, whereas conspicuous species should be conspicuous on most backgrounds. If we used an average of all backgrounds, species that have a bimodal distribution (i.e., easy to find on some backgrounds and hard to find on others) could have the same detection time than a species that have a mid‐range detection on all backgrounds. That way, our detection time data are equivalent to measuring detection time of a white rabbit against a white background and a brown rabbit against a brown background. The detection time for each butterfly was measured in seconds and varied from highly conspicuous (0 s, immediate detection) to highly cryptic (10 s or failure to detect).

To determine if the dorsal and ventral wing sides in toxic butterflies had similar detection times than in nontoxic butterflies, we calculated the time difference by subtracting the mean dorsal time from mean ventral time on all 30 backgrounds. Then we calculated the absolute mean of that time difference.

## 3 Analyses

We used two Bayesian multilevel models from the ‘brms’ package (Bürkner 2018) to test our hypotheses. For the first hypothesis (does detectability predict toxicity) we related toxicity to dorsal and ventral detectability against the most cryptic background. Predictors (ventral detection time and dorsal detection time) were not collinear (vif < 2, Fox & Weisberg, 2019). For the species which had no dorsal image (*n* = 5) we used the mean of dorsal detection time of all species, allowing us to include dorsal and ventral detection time in the same model without impacting the outcome. To assure that including these five species did not affect the results, we created a separate model removing species with missing values, and results remained the same (Table S2). Then, we standardised predictors using z‐scores, so that each value is expressed as the number of standard deviations from the mean detection time. The response variable was *Daphnia* mortality, modelled as the number of deaths relative to the total number of individuals tested in each assay (deaths | trials(N.daphnia)), where *deaths* is the number of dead *Daphnia* and *N.daphnia* is the total number of individuals in each assay. To control for nonindependence among closely related species we included phylogeny as a random factor in the model. This measure was achieved by cropping the species closest to our samples from the most recent butterfly phylogeny (Kawahara et al. 2023) and calculating the correlation matrix between species using the package ‘ape’ (Paradis et al. 2024). Our predictors had flat priors, and the phylogenetic matrix had a student's prior. We calculated the effect of our model by using bayes_R2 from ‘brms’ (Bürkner 2018). Additionally, we used the performance package to check how much of the R^2^ is attributed to the fixed effect (Lüdecke et al. 2021). The second Bayesian multilevel model followed the same procedure, but the response variable was absolute mean time difference (calculated as described above).

Analyses were conducted in R version 4.4.0 (R Core Team 2024). All data are available from GitHub (http://github.com/MFErickson/Does‐conspicuousness‐relate‐to‐toxicity‐in‐butterflies).

## 4 Results

Control assays showed negligible mortality. The solvent controls produced similarly low mortality (median = 0; mean mortality: Milli‐Q water = 0.00463, methanol = 0.00556), with the maximum mortality observed in a single assay being one *Daphnia*. Extracts from the reference organisms *Acheta domesticus* and *Nauphoeta cinerea* also showed very low mortality (mean = 0.03 for both), with a maximum of two *Daphnia* killed in a single assay. These results indicate that the mortality observed in butterfly extracts was unlikely to be caused by residual solvent and instead reflected chemicals present in the butterflies.

The backgrounds against which butterflies were most difficult to detect varied among species, although some substrates occurred more frequently than others. On the dorsal side, moss backgrounds were commonly identified as the most cryptic, including moss2 (15 species) and moss1 (10 species). Other commonly cryptic backgrounds included litter1 (10 species) and flowers1 (9 species). A similar pattern was observed for ventral surfaces. Leaf litter background was the most frequently associated with the longest detection times, particularly litter1 (17 species), followed by moss backgrounds moss2 (9 species) and moss1 (8 species). Easiest to detect backgrounds also varied among species. On both sides, butterflies were more easily detected against the flower3 background (Dorsal = 8, Ventral = 14), followed by grass2 (Dorsal = 7, Ventral = 8) and smallrock1 (Dorsal = 7 and Ventral = 8). Several additional backgrounds were identified as the most cryptic and most conspicuous for smaller numbers of species, indicating substantial variation in which substrates provided the greatest concealment.

Ventral detection time on the most cryptic background varied from 3.4 to the maximum allowed time of 10 s (Figure 1), while dorsal detection time varied from 2.5 to 10 s (Figure 2). For the human observer, there appeared to be a gradient whereby ventral butterflies that appear lighter in colour (see Figure 1) were detected more quickly. Visual inspection of the dorsal images suggested greater variability in colour patterns than ventral images, with blue butterflies appearing to be detected more quickly, followed by most, but not all, white and yellow butterflies. (Figure 2).

**FIGURE 4:**
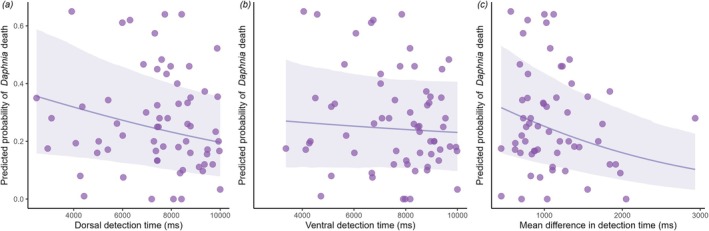
Proportion of *Daphnia* mortality against (a) dorsal and (b) ventral detection time (c) mean absolute detection time difference of all possible backgrounds. Light purple line represents predicted probability of *Daphnia* mortality and shaded areas represent the 95% credible interval.

**FIGURE 5:**
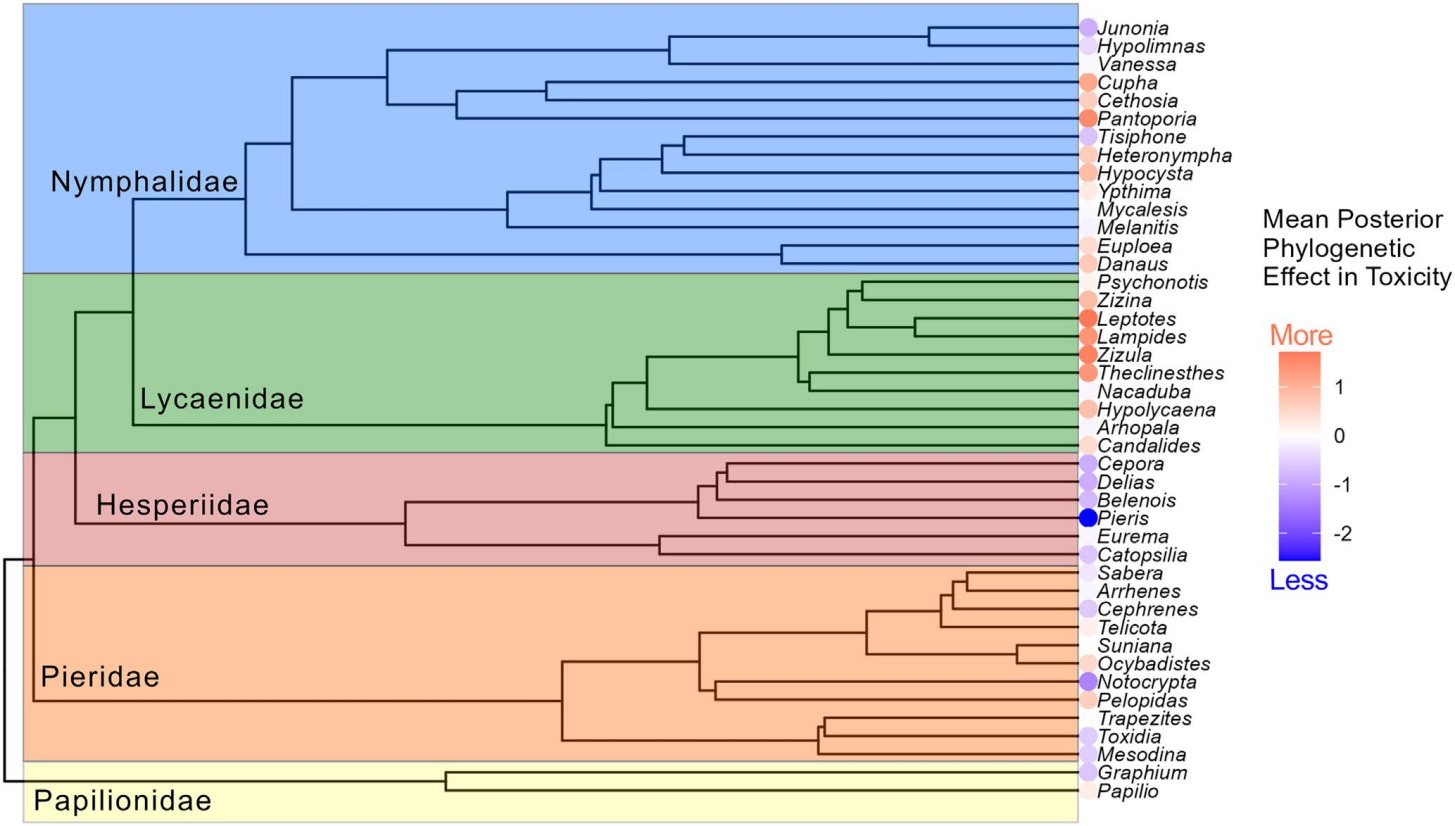
Mean posterior effect of genus on butterfly toxicity (according to brms model). Dots indicate strength of effect where redder dots indicated a stronger positive effect, and bluer dots represent a stronger negative effect on toxicity. Tree file extracted from Kawahara et al. (2023), and trimmed to relevant genus (as opposed to species that was calculated in the model) to improve visibility.

Dorsal detection time was negatively related to *Daphnia* mortality, meaning that more conspicuous species (i.e., with faster detection times) were more toxic. Ventral detection time, however, was not significantly related to *Daphnia* mortality (Table 1). Our full model predicted 86% of the variation in *Daphnia* mortality (Bayes R^2^ = 0.86), with ventral and dorsal detection time contributing a quarter (marginal R^2^ = 0.246) of the variation in toxicity (Figure 4) and phylogeny responsible for the remaining variation. *Leptotes*, *Zizula and Pantoporia* had the largest positive effect on toxicity, whereas *Pieris*, *Notocrypta* and *Delias* had the strongest negative impact on toxicity (Figure 5). Mean absolute detection difference between dorsal and ventral side was a significant predictor of toxicity, with butterflies that had more similar detection time (between the two sides) being more likely to be toxic (Bayes R^2^ = 0.87, marginal R^2^ = 0.282).

**TABLE 1:** Output of Bayesian multilevel model of log‐odds of *Daphnia* death (toxicity).

Model 1	Estimate	Est.Error	l‐95% CI	u‐95% CI	Rhat	Bulk_ESS	Tail_ES
Intercept	−1.18	0.45	−2.11	−0.34	1	1496	1493
**Dorsal time**	**−0.21**	**0.1**	**−0.42**	**−0.03**	**1**	**1452**	**1609**
Ventral time	−0.06	0.1	−0.26	0.15	1	1774	1534
Model 2							
Intercept	−1.2	0.44	−2.04	−0.32	1	1441	1495
**Mean time difference**	**−0.27**	**0.08**	**−0.43**	**−0.12**	**1**	**1729**	**1380**

*Note:* Model 1 uses absolute mean dorsal–ventral detectability difference of most cryptic background as predictor; Model 2 uses the absolute mean of the time difference of dorsal minus ventral detection time of each of the 30 backgrounds. Estimate represents the posterior mean, with Est. Error indicating the standard error estimate. The l‐95% CI and u‐95% CI provide the lower (l) and upper (u) bounds of the 95% credible interval. Rhat is the Gelman‐Rubin diagnostic for MCMC convergence, where Rhat = 1 indicates good convergence. Bulk_ESS represents the effective sample size for estimating means and standard deviations, while Tail_ESS captures the effective sample size for the tails of the posterior distribution. Significant predictor effects (parameters whose 95% credible intervals do not overlap zero) are shown in bold.

## 5 Discussion

Overall, we found evidence to link human detection time to toxicity in butterflies: Butterflies that were easier to detect on their dorsal side were more likely to be toxic, but contrary to our prediction, we did not find a similar pattern for the ventral wing surface. As predicted, we also found that species that had similar detection times for dorsal and ventral surfaces against the same background were more likely to be toxic, meaning that toxic butterflies tended to be similarly detectable regardless of whether they are exposing their dorsal or ventral side. These results may seem contradictory, but they simply mean that despite toxic species having more consistent signals on both sides, the dorsal side is salient. We note that our measures of detection time represent different aspects in the two analyses. In the first analysis, which tests whether species toxicity is related to detection time, we used the most cryptic background separately for dorsal and ventral sides; these backgrounds were often not the same. In the second analysis, which examines whether dorsal and ventral sides are similarly detectable, we compared detection times against the same background. These differences may arise because visually distinct patterns on the dorsal and ventral surfaces are differentially salient, providing better camouflage against specific backgrounds, while still producing similar detection times when viewed against the same background.

Turning to the phylogenetic distribution of toxicity, which was not a focus of this study, we found a strong phylogenetic component in toxicity. Nymphalids and lycaenids (Kawahara et al. 2023), which are sister clades, contained most genera that had a positive effect on toxicity (Lycaenidae 8/10, Nymphalidae 8/14, Papilionidae 1/2, Hesperiidae 3/11 and Pieridae 0/6). Of these, nymphalids, such as monarchs, are classic models in the study of evolution of aposematism, whereas lycaenids are more neglected, probably because of their small size and because most species have blue hues, which are usually not considered to be aposematic (but see Erickson 2025). Although we did not have any Riodinidae butterflies, they would be an interesting comparison group as a sister group to lycaenids and thus a potential target for toxin discovery.

In our study, we measured detection time as a proxy for conspicuousness based on several studies that measured conspicuousness via contrast values and found a strong relationship with detection time (Bohlin et al. 2008; Stevens et al. 2013; Tullberg et al. 2005). Butterflies that were more likely to be toxic were detected faster on the dorsal side. Although both dorsal and ventral wing surfaces are exposed during flight due to wing flapping, butterflies are rarely viewed dorsally while resting, as most species rest with their wings closed exposing the ventral surface. Basking, sitting with both wings open, represents a distinct behavioural state in which butterflies warm their flight muscles in preparation for take‐off (Barton et al. 2014). Under these conditions, individuals with conspicuous dorsal coloration may be easier to detect but are physiologically ready for rapid escape once detected. Thus, higher detectability on the dorsal side is consistent with the idea that conspicuous and toxic species may better tolerate detectability during motion (Speed et al. 2010) or immediate escape, when aposematic signals and flight can jointly reduce predation risk. Concurrently, less conspicuous, cryptic patterns may be more advantageous on the ventral wing surface, which is exposed when butterflies rest with their wings closed and are less mobile, and therefore more vulnerable to predation (Hall et al. 2013).

With conspicuousness of toxic butterflies being more prominent on the dorsal side than on the ventral side, there is the opportunity for the evolution of independent colour defences, a hypothesis known as ‘signal partitioning’ (Wee and Monteiro 2017). This is best illustrated by butterflies and insects that utilise a startle display: their ventral side is often cryptically coloured, but when they open their wings, bright colours or eyespots are revealed (see Umbers et al. 2018). Work on mimicry rings in brush‐footed butterflies suggests that ventral, rather than dorsal, wing coloration plays a primary role in mate recognition and selection, whereas the dorsal surface functions primarily as an aposematic signal (Dell'Aglio et al. 2018). This pattern may arise because brush‐footed butterflies have evolved visual systems that are particularly sensitive to ventral wing colour patterns (Dell'Aglio et al. 2018), and is further highlighted by their characteristic mating displays, which expose the ventral surface during courtship (Klein and de Araújo 2010). In our study, we did not test for the presence of mimics among our species but distinguishing between model and mimic species in future work could further explain the relationship between toxicity and conspicuousness, which is clearly complex.

We found that toxic species were more likely to have similar dorsal and ventral detection times, which aids recognition by predators regardless of viewer angle and butterfly posture. Yet ventral detection was not significantly related to toxicity, suggesting that there may be a benefit to not signalling strongly while resting and exposing the ventral surface. Sexual selection may work concomitantly with aposematic signals on the dorsal side (Maisonneuve and Aubier 2025). Indeed, dorsal wing surfaces are considered to be important for sexual signals (Chappell et al. 2023; Oliver et al. 2009; White et al. 2015). To date, there are only a few well‐studied systems that link sexual and aposematic signal design, such as the wood tiger moth, *Arctia plantaginis* (Rojas et al. 2018), in which there is an apparent trade‐off between aposematic colouration and mate attraction (Nokelainen et al. 2011).

Detectability arises from an interaction between the colouration of the target (here, butterflies), the visual system of the observer (in this study, humans), ambient light conditions, and the background against which the target is viewed. Butterfly colour patterns have evolved within specific ecological contexts, and no single pattern is optimal across all environmental conditions. For instance, camouflage can be more effective than aposematism at low light levels (Medina et al. 2025). In our study, we therefore assumed that aposematic species are likely to remain relatively conspicuous across multiple backgrounds, whereas cryptic species are optimised for camouflage in a narrow range of backgrounds. In natural settings, however, both aposematic and non‐aposematic species are likely to encounter suboptimal backgrounds during their lifetime, which may increase predation pressure and drive selective shifts in colouration. For example, deforestation in the Amazon rainforest has been associated with less colourful butterfly communities, potentially because nondefended species become more detectable in degraded habitats due to increased background contrast (Spaniol et al. 2020). Predator community composition may further influence colour evolution (Kikuchi et al. 2021): if predators resistant to chemical defences are abundant, selection may favour reduced detectability even in chemically defended species. Similarly, under high overall predation pressure, reduced detectability may be advantageous even for toxic species. Consistent with this, predator avoidance learning can be enhanced either through increased conspicuousness or increased toxicity, indicating that conspicuousness is not the only mechanism of aposematism (Darst et al. 2006).

While many aposematic colour signals are conspicuous and generate strong internal and external contrasts, aposematism can also work when predators can distinguish between toxic and non‐toxic prey by characteristics other than conspicuousness (Endler and Mappes 2004; Merilaita and Ruxton 2007; Sherratt and Beatty 2003), although it is acknowledged that distinctiveness is more easily achieved through conspicuousness. Despite undefended prey being predicted to be cryptic (Merilaita and Ruxton 2007), this does not stop cryptic species from being defended (see also Endler and Mappes 2004; Gonzalez et al. 2021). Indeed, we found toxic species that took a long time to detect. This might be because some butterfly predators are not visual hunters or because of the diverse functions of toxins, such as deterrents of parasitism (de Roode and Groot 2024) or precursors of sex pheromones (Eisner and Meinwald 1995). Thus, the visual traits necessary to elicit aversive learning, and therefore aposematism, are likely to be complex and are still poorly known (Stevens and Ruxton 2012).

We recognise there are several limitations of our study. First, toxins isolated are dependent on the solvent used, and hence our method likely did not isolate all possible chemicals that can serve as toxins. For a more comprehensive isolation of toxins, multiple solvents could be used, but this requires a much larger number of samples. Ideally, toxins should be tested against naturally occurring predators to corroborate their functions as antipredator defence (Rojas et al. 2017; Rubiano‐Buitrago et al. 2024). Another avenue of identification of toxic chemicals could be chromatography, which would allow for a more biologically meaningful assessment of toxicity. Second, conspicuous signals can signal nontoxic defences (e.g., flight speed; Ottocento et al. 2023; Páez et al. 2021), which could have underestimated the number of species that are defended. Third, our human proxy predators cannot perceive UV colours that are common in butterfly wings and visible to many visual hunting predators, so the detectability results do not reflect those of real predators. Researchers are now developing games that can be used by actual predators, which could mediate this limitation in the future (Silvasti et al. 2024). Lastly, we limited the detection time to 10 s and the *Daphnia* mortality to 10 *Daphnia* per assay. This may introduce ceiling effects, that make more cryptic or toxic species appear less so. Nevertheless, here we attempted to go beyond computational models and opted for a large‐scale study that relates classic aposematic traits (detectability as a proxy for conspicuousness and toxicity). This is one of the first studies that attempts to do so, and we urge more research linking multiple antipredatory traits such as toxicity and colour patterns, especially at community levels.

## Author Contributions

**Marilia Fernandes Erickson:** conceptualization (equal), data curation (equal), formal analysis (lead), investigation (lead), methodology (equal), project administration (lead), visualization (lead), writing – original draft (lead), writing – review and editing (equal). **Donald James McLean:** conceptualization (equal), data curation (equal), formal analysis (equal), methodology (equal), resources (equal), software (lead), visualization (supporting), writing – review and editing (equal). **Marie E. Herberstein:** conceptualization (equal), funding acquisition (lead), methodology (equal), resources (equal), supervision (lead), writing – review and editing (equal).

## Funding

This work was supported by Macquarie University, International Macquarie Research Excellence Scholarship and by Australian Research Council, DP220102323.

## Conflicts of Interest

The authors declare no conflicts of interest.

## Supporting information


**Table S1:** Percentage of average *Daphnia* mortality per species.

**Table S2:** Output of Bayesian Multilevel Model of log‐odds of *Daphnia* death (toxicity). This model excludes 5 species (*Mesodina halyzia*, *Nacaduba berenice*, *Ocybadistes flavovittatus*, *Pelopidas lyelli* and *Sabera fuliginosa*) where we only had ventral images and hence does not replace missing values with the mean. This model predicts a stronger effect of dorsal detection time on toxicity. Estimate represents the posterior mean, with Est. Error indicating the standard error estimate. The l‐95% CI and u‐95% CI provide the lower (l) and upper (u) bounds of the 95% credible interval. Rhat is the Gelman‐Rubin diagnostic for MCMC convergence, where RHAT = 1 indicates good convergence. Bulk_ESS represents the effective sample size for estimating means and standard deviations, while Tail_ESS captures the effective sample size for the tails of the posterior distribution.

## Data Availability

Part of the toxicity data in this project is shared with Erickson et al. (in prep) and Erickson et al. (2025). Photographs used to generate butterfly images for the game were obtained from Erickson et al. (2025). All raw data obtained for this manuscript and processed data from the listed sources used in this project are found in the following GitHub repository: https://github.com/MFErickson/Does‐conspicuousness‐relate‐to‐toxicity‐in‐butterflies.
