# Correction to “Are Toxic Butterflies More Easily Detected by Human ‘Predators’?”

**DOI:** 10.1002/ece3.74167

**Published:** 2026-08-07

Erickson, M. F. , D. J. McLean , and M. E. Herberstein . 2026. “Are Toxic Butterflies More Easily Detected by Human ‘Predators’?” Ecology and Evolution 16, no. 4: e73357. 10.1002/ece3.73357.41948209 PMC13053117

In Figure 5 of the originally published version of this article, the labels “Hesperiidae” and “Pieridae” were mistakenly switched. The correct Figure 5 is displayed below.

**FIGURE 5:**
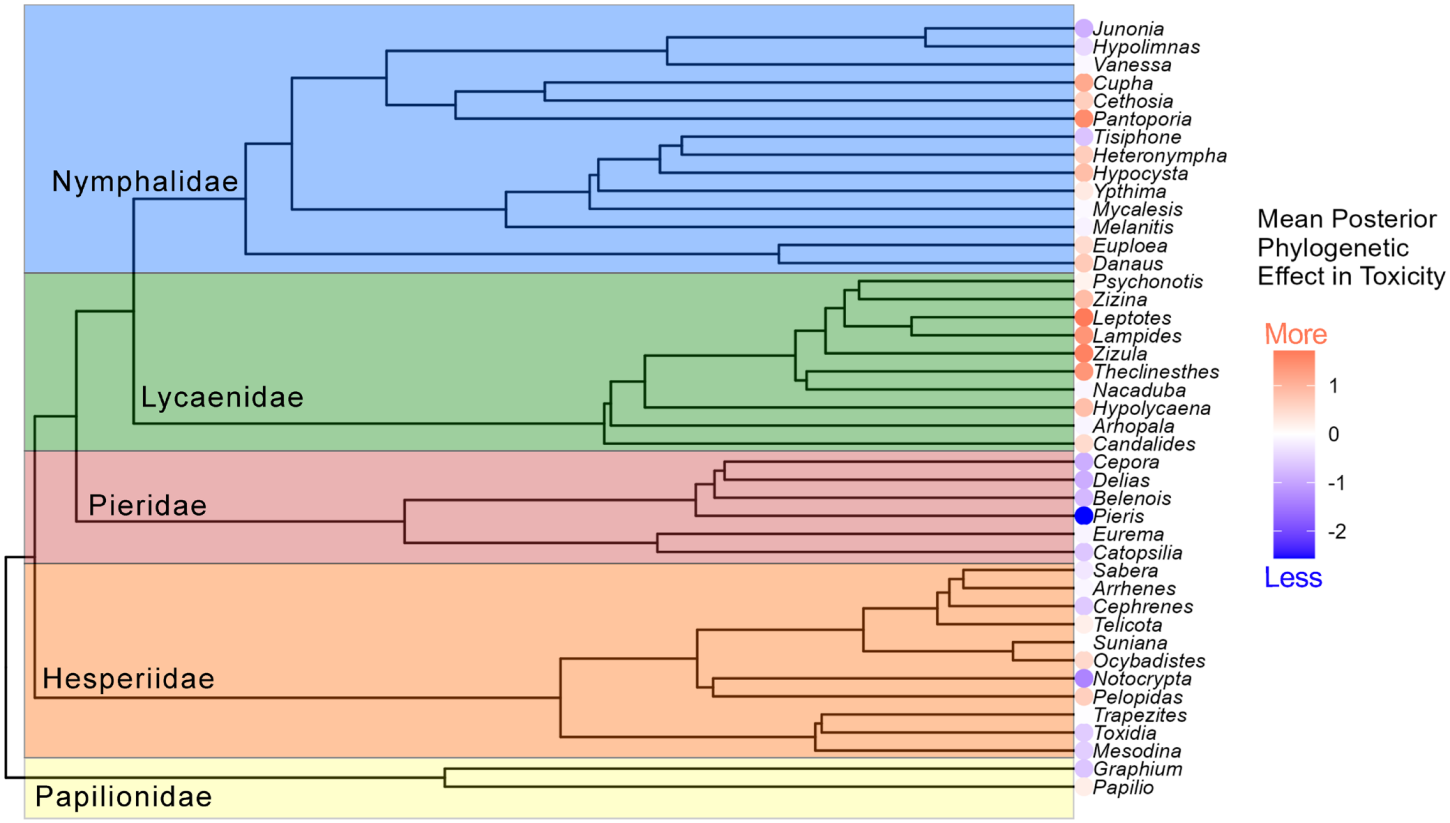
Mean posterior effect of genus on butterfly toxicity (according to brms model). Dots indicate strength of effect where redder dots indicated a stronger positive effect, and bluer dots represent a stronger negative effect on toxicity. Tree file extracted from Kawahara et al. (2023), and trimmed to relevant genus (as opposed to species that was calculated in the model) to improve visibility.

We apologize for this error.

