# Supplementary material for: Are Toxic Butterflies More Easily Detected by Human ‘Predators’?
Source: Ecol Evol. 2026 Apr 6;16(4):e73357. doi: 10.1002/ece3.73357 (PMC13053117; doi:10.1002/ece3.73357)
Supplement: Supplementary file 1 — Table S1: Percentage of average Daphnia mortality per species. [file ECE3-16-e73357-s001.docx]

Table s1. Percentage of average *Daphnia* mortality per species.

| Family | Species | *Daphnia* mortality (%) |
| --- | --- | --- |
| Hesperiidae | *Arrhenes dschilus* | 0.166 |
|  | *Cephrenes augiades* | 0.033 |
|  | *Mesodina halyzia* | 0.133 |
|  | *Notocrypta waigensis* | 0 |
|  | *Ocybadistes flavovittatus* | 0.441 |
|  | *Ocybadistes walkeri* | 0.233 |
|  | *Pelopidas agna* | 0.354 |
|  | *Pelopidas lyelli* | 0.25 |
|  | *Sabera caesina* | 0.171 |
|  | *Sabera fuliginosa* | 0.133 |
|  | *Suniana sunias* | 0.155 |
|  | *Telicota ancilla* | 0.111 |
|  | *Telicota mesoptis* | 0.352 |
|  | *Toxidia peron* | 0.12 |
|  | *Trapezites symmomus* | 0.2 |
| Lycaenidae | *Arhopala micale* | 0.28 |
|  | *Candalides absimilis* | 0.65 |
|  | *Candalides hyacinthinus* | 0.12 |
|  | *Hypolycaena phorbas* | 0.466 |
|  | *Lampides boeticus* | 0.572 |
|  | *Leptotes plinius* | 0.64 |
|  | *Nacaduba berenice* | 0.325 |
|  | *Nacaduba cyanea* | 0.175 |
|  | *Psychonotis caelius* | 0.35 |
|  | *Theclinesthes onycha* | 0.611 |
|  | *Zizina otis* | 0.415 |
|  | *Zizula hylax* | 0.64 |
| *Nymphalidae* | *Cethosia cydippe* | 0.333 |
|  | *Cupha prosope* | 0.483 |
|  | *Danaus affinis* | 0.62 |
|  | *Danaus petilia* | 0.166 |
|  | *Danaus plexippus* | 0.33 |
|  | *Euploea corinna* | 0.25 |
|  | *Euploea tulliolus* | 0.46 |
|  | *Heteronympha merope* | 0.46 |
|  | *Heteronympha mirifica* | 0.2 |
|  | *Hypocysta metirius* | 0.372 |
|  | *Hypolimnas bolina* | 0.15 |
|  | *Junonia hedonia* | 0.1 |
|  | *Junonia villida* | 0.09 |
|  | *Melanitis leda* | 0.18 |
|  | *Mycalesis perseus* | 0.16 |
|  | *Mycalesis sirius* | 0.25 |
|  | *Mycalesis terminus* | 0.181 |
|  | *Pantoporia consimilis* | 0.522 |
|  | *Tisiphone abeona* | 0.093 |
|  | *Vanessa kershawi* | 0.2 |
|  | *Ypthima arctous* | 0.26 |
| Papilionidae | *Graphium choredon* | 0.187 |
|  | *Papilio aegeus* | 0.28 |
|  | *Papilio demoleus* | 0.28 |
| Pieridae | *Belenois java* | 0.170 |
|  | *Catopsilia pomona* | 0.2 |
|  | *Cepora perimale* | 0.16 |
|  | *Delias argenthona* | 0.2 |
|  | *Delias mysis* | 0.22 |
|  | *Delias nigrina* | 0 |
|  | *Eurema hecabe* | 0.342 |
|  | *Eurema laeta* | 0.075 |
|  | *Eurema smilax* | 0.261 |
|  | *Pieris rapae* | 0.01 |
