# Supplementary material for: Are Toxic Butterflies More Easily Detected by Human ‘Predators’?
Source: Ecol Evol. 2026 Apr 6;16(4):e73357. doi: 10.1002/ece3.73357 (PMC13053117; doi:10.1002/ece3.73357)
Supplement: Supplementary file 2 — Table S2: Output of Bayesian Multilevel Model of log‐odds of Daphnia death (toxicity). This model excludes 5 species (Mesodina halyzia, Nacaduba berenice, Ocybadistes flavovittatus, Pelopidas lyelli and Sabera fuliginosa) where we only had ventral images and hence does not replace missing values with the mean. This model predicts a stronger effect of dorsal detection time on toxicity. Estimate represents the posterior mean, with Est. Error indicating the standard error estimate. The l‐95% CI and u‐95% CI provide the lower (l) and upper (u) bounds of the 95% credible interval. Rhat is the Gelman‐Rubin diagnostic for MCMC convergence, where RHAT = 1 indicates good convergence. Bulk_ESS represents the effective sample size for estimating means and standard deviations, while Tail_ESS captures the effective sample size for the tails of the posterior distribution. [file ECE3-16-e73357-s002.docx]

**Table s2.** Output of Bayesian Multilevel Model of log-odds of *Daphnia* death (toxicity). This model excludes 5 species (*Mesodina halyzia*, *Nacaduba berenice*, *Ocybadistes flavovittatus*, *Pelopidas lyelli* and *Sabera fuliginosa*) where we only had ventral images and hence does not replace missing values with the mean. This model predicts a stronger effect of dorsal detection time on toxicity. Estimate represents the posterior mean, with Est. Error indicating the standard error estimate. The l-95% CI and u-95% CI provide the lower (l) and upper (u) bounds of the 95% credible interval. Rhat is the Gelman-Rubin diagnostic for MCMC convergence, where RHAT = 1 indicates good convergence. Bulk_ESS represents the effective sample size for estimating means and standard deviations, while Tail_ESS captures the effective sample size for the tails of the posterior distribution.

| Model evo (exclude ventral only) |  |  |  |  |  |  |  |
| --- | --- | --- | --- | --- | --- | --- | --- |
|  | Estimate | Est.Error | l-95% CI | u-95% CI | Rhat | Bulk_ESS | Tail_ES |
| Intercept | -1.22 | 0.53 | -2.31 | -0.13 | 1 | 1467 | 1422 |
| **Dorsal time** | **-0.34** | **0.14** | **-0.63** | **-0.08** | **1** | **1442** | **1613** |
| Ventral time | -0.25 | 0.14 | -0.53 | 0.03 | 1 | 1399 | 1534 |
